# Reversible Gynecomastia in HIV-Infected Man Treated With Triple Antiretroviral Therapy Containing Efavirenz: A Case Report

**DOI:** 10.7759/cureus.27991

**Published:** 2022-08-14

**Authors:** Mohammed Amine Essafi, Lamiaa Elazizi, Hayat Aynaou, Houda Salhi, Hanan El Ouahabi

**Affiliations:** 1 Department of Endocrinology, Diabetology, Metabolic Diseases and Nutrition, Hassan II University Hospital Center, Fez, MAR

**Keywords:** efavirenz., antiretroviral therapy, hiv, reversible gynecomastia, bilateral gynecomastia

## Abstract

Gynecomastia is benign hypertrophy of male breast glandular tissue, either unilateral or bilateral, secondary to increased estrogen/testosterone ratio (elevated estrogen level, decreased testosterone levels, or both). The condition can be related to a medical disease or caused by some drugs.

Since the introduction of triple antiretroviral therapy (TAT), we have seen an improvement in the prognosis of human immunodeficiency virus (HIV) infection.

Here we report the case of a 53-year-old man receiving follow-up care in Internal Medicine for HIV infection receiving TAT (tenofovir/efavirenz/emtricitabine). After one year, the patient presented in the Department of Endocrinology, Diabetology, Metabolic Diseases, and Nutrition of Hassan II University Hospital Center, Fez, with bilateral gynecomastia. Hormonal exploration did not reveal any abnormality, so the gynecomastia was attributed to efavirenz use. The regimen was replaced by tenofovir, lamivudine, and dolutegravir. The gynecomastia was resolved within two months of discontinuing efavirenz.

In summary, we think that secondary gynecomastia should be suspected and screened in HIV patients receiving efavirenz-containing regimens.

## Introduction

Gynecomastia is defined as benign proliferation of the glandular tissue of the male breast [1]. The diagnosis is made clinically, and excess adipose tissue or pseudogynecomastia is the main differential diagnosis.

Several drug classes have been flagged as having a side effect of gynecomastia [2], including antiretrovirals. Currently, antiretroviral treatment is indicated for all HIV-positive people, and most prescribed regimens contain efavirenz, as recommended by the World Health Organization [3].

Gynecomastia has been described as a side effect of triple antiretroviral therapy (TAT), but its mechanism remains unclear. Although gynecomastia is benign, it has significant psychological effects that may impact treatment adherence and success. Some cases of gynecomastia have been reported in HIV-infected men treated with TAT.

We report the case of a 53-year-old man who developed gynecomastia while on efavirenz-containing TAT and recovered two months after discontinuation of efavirenz and change in regimen.

## Case presentation

The 53-year-old man was followed up in internal medicine for HIV for one year on TAT (tenofovir/efavirenz/emtricitabine) with hypothyroidism post-thyroidectomy four years prior under substitution and operated on for a left breast nodule one-and-half years prior with benign histology. The patient was referred to the Department of Endocrinology, Diabetology, Metabolic Diseases, and Nutrition for the investigation and management of bilateral gynecomastia. The onset of the symptomatology dated back four months (eight months after beginning TAT) when the patient noticed a progressive increase in the volume of the breasts (more marked on the right side) associated with pain on palpation, without perception of nodule or nipple discharge, and with notions of decreased libido and severe erectile dysfunction.

**Figure 1:**
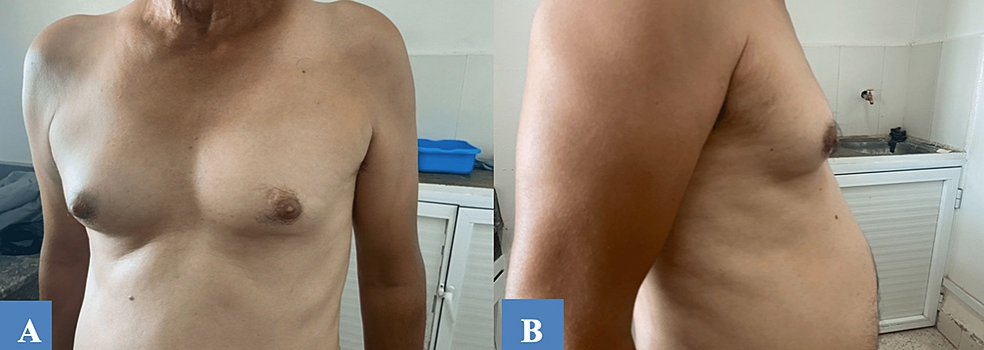
Patient’s (A) face and (B) profile show gynecomastia that is more marked on the right side.

Upon clinical examination, the patient was found in good general health, with gynecomastia grade I on the left glandular and grade IIa on the right glandular, which was tender to palpation without perception of nodule or nipple discharge, or axillary adenopathy (Figure 1).

The patient’s scrotum was wrinkled and pigmented, with palpation of a firm extra testicular nodule on the left side measuring 2.5 cm on the long axis. The two testicles were intra-scrotal, measuring 6 cm x 4 cm on the right side and 5.5 cm x 4 cm on the left side, indicative of Tanner stage V. The penis measured 8.5 cm x 2.5 cm with no hypospadias.

Gynecomastia was confirmed by echomammography, with aspects of bilateral gynecomastia in its dendritic form more marked on the right side and with a heterogeneous hyperechoic zone in the external quadrant of the left breast most likely related to postoperative remodeling. To eliminate obvious causes, TSH and liver and kidney function were normal, as were hormonal (Testosterone, eostradiol, FSH, LH Prolactin) and tumor markers (Alpha-fetoprotein (AFP) and bHCG). A testicular ultrasound found a left epididymal cyst measuring 52.5 mm x 28.7 mm with a right hydrocele blade, and the patient was referred to urology.

**Figure 2:**
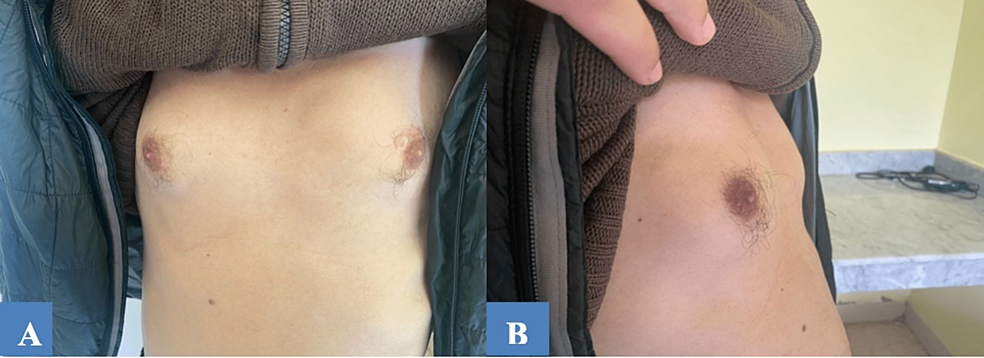
Patient’s (A) face and (B) profile showing a regression of gynecomastia two months after discontinuing efavirenz.

After discussing with the department of pharmacovigilance, the gynecomastia was attributed to efavirenz use. The regimen was replaced by tenofovir, lamivudine, and dolutegravir (TLD) by the Department of Internal Medicine. The breast pain and gynecomastia resolved within two months of stopping efavirenz (Figure 2).

## Discussion

Gynecomastia is a frequently encountered condition that can be caused by some drugs, and an algorithm for its management has been developed [4].

A few studies have estimated the prevalence of HIV-associated gynecomastia to be approximately 1.8%-3% [5-6].

Currently, antiretroviral treatment is indicated for all HIV-positive people [3]. Efavirenz is a non-nucleoside reverse transcriptase inhibitor that is present in most prescribed first-line HIV regimens [7]. Gynecomastia has been recognized as a side effect of efavirenz, which should be distinguished from lipodystrophy syndrome caused by nucleoside reverse transcriptase inhibitors as part of TAT.

In our case report, gynecomastia was attributed to efavirenz use. Some case reports and small case series have reported gynecomastia as a side effect of efavirenz [8-13]; the physio-pathological mechanism is unclear, but there are some hypotheses about estrogen hormone activation [11,14,15]. In an experimental study by Sikora et al. [11] direct estrogenic effect of efavirenz was explained. In their study, it was proved that efavirenz can induce the growth of breast cancer cell lines MCF-7 and ZR-75-1, which have estrogen receptor (ER) positivity. It was also proved that efavirenz can directly bind to ER-alpha (ER-a). These effects were observed in the cell model at the dose of 600 mg of daily administration of efavirenz.

In the literature, the reported cases were bilateral and resolved upon discontinuation of efavirenz, similar to our patient (Table 1) [9,10,12,13,16,17]. The time of resolution in our case report was two months after the discontinuation of efavirenz, which is comparable to other studies that have reported a resolution time of three to 10 months [9,10,12,13,16,17].

**Table 1:** Cases in the literature of efavirenz-induced gynecomastia.

	Age	Gynecomastia	Latent period (months)	Time to resolution (months)
Kwekwesa et al. [9]	35-year-old male	Bilateral	12	6
	56-year-old male	Bilateral	60	6
Njugunam et al. [10]	51 cases of efavirenz	Bilateral 57%	15 (median)	3 (median)
Kratz et al. [12]	32-year-old male	Bilateral	24	9
	68-year-old male	Bilateral	2	12
Oche et al. [13]	34 years	Bilateral	11	-
	44 years	Bilateral	8	6
	51 years	Unilateral	8	4
	42 years	Unilateral	9	8
	38 years	Bilateral	13	6
	40 years	Bilateral	16	10
Shawarira-Bote et al. [16]	22 cases	58% Bilateral	24 (median)	3 (median)
Jover et al. [17]	30	Bilateral	8	5
	36	Unilateral	7	5
	39	Unilateral	4	5
	37	Unilateral	5	5
	47	Unilateral	15	5
Our case report	53 years	Bilateral	8	2

Gynecomastia can have significant psychological side effects that can impact treatment adherence and success. It is important to inform patients of this side effect and encourage consultation at the onset of gynecomastia. Similarly, clinicians should systematically look for this side effect during follow-up and manage it promptly.

Early discontinuation of efavirenz may be associated with rapid and complete resolution of gynecomastia. A late diagnosis, especially at the fibrosis stage, can lead to persistent gynecomastia even after stopping Efavirenz.

## Conclusions

Our case report shows gynecomastia secondary to efavirenz with resolution after discontinuation. It is important to inform patients of this side effect and to encourage clinicians to look for it for early management before the fibrosis stage. Active screening for gynecomastia in men on antiretroviral therapy is recommended for possible early discontinuation of efavirenz to improve treatment adherence and success.
